# Diagnostic accuracy of pleural fluid lactate dehydrogenase to adenosine deaminase ratio for tuberculous pleural effusion: an analysis of two cohorts

**DOI:** 10.1186/s12890-022-02247-w

**Published:** 2022-11-19

**Authors:** Zhi Yan, Jian-Xun Wen, Hua Wang, Ting-Wang Jiang, Jin-Hong Huang, Hong Chen, Li Yan, Zhi-De Hu, Wen-Qi Zheng

**Affiliations:** 1 Department of Laboratory Medicine, The Affiliated Hospital of Inner Mongolia Medical University, Tongdao North Street 1, Hohhot, 010050 China; 2 Department of Parasitology, The Basic Medical Sciences College of Inner Mongolia Medical University, Hohhot, 010050 China; 3 Department of Medical Experiment Center, The Basic Medical Sciences College of Inner Mongolia Medical University, Hohhot, 010050 China; 4 Department of Key Laboratory, The Affiliated Changshu Hospital of Xuzhou Medical University, Suzhou, 215500 China; 5 Department of Pulmonary and Critical Care Medicine, The Affiliated Changshu Hospital of Xuzhou Medical University, Suzhou, 215500 China; 6 Department of Respiratory and Critical Care Medicine, The Affiliated Hospital of Inner Mongolia Medical University, Hohhot, 010050 China

**Keywords:** Lactate dehydrogenase, Adenosine deaminase, Tuberculous pleural effusion, Diagnostic accuracy, Added value

## Abstract

**Background:**

This study aimed to evaluate the diagnostic accuracy of pleural fluid (PF) lactate dehydrogenase (LDH) to adenosine deaminase (ADA) (LDH/ADA) ratio for tuberculous pleural effusion (TPE). Especially to explore whether the LDH/ADA ratio provides added diagnostic value to ADA.

**Methods:**

The diagnostic accuracy of PF LDH/ADA ratio and ADA for TPE was evaluated in two cohorts, named the BUFF (Biomarkers for patients with Undiagnosed pleural eFFusion) cohort (62 with TPE and 194 with non-TPE) and the SIMPLE (a Study Investigating Markers in PLeural Effusion) cohort (33 with TPE and 177 with non-TPE). Receiver operating characteristic (ROC) curve and decision curve were used to measure the diagnostic accuracy of the PF LDH/ADA ratio. The added diagnostic value of the LDH/ADA ratio to ADA was evaluated with net reclassification improvement (NRI) and integrated discrimination improvement (IDI).

**Results:**

The area under the ROC curves (AUCs) of PF ADA and LDH/ADA ratio in the BUFF cohort were 0.76 and 0.74, respectively. In the SIMPLE cohort, the AUCs of PF ADA and LDH/ADA ratio were 0.80 and 0.85, respectively. The decision curves of PF LDH/ADA and ADA were close in both the BUFF and SIMPLE cohorts. The NRI and IDI analyses did not reveal any added diagnostic value of LDH/ADA to ADA.

**Conclusions:**

PF LDH/ADA ratio has moderate diagnostic accuracy for TPE. It does not provide added diagnostic value beyond ADA. The current evidence does not support LDH/ADA ratio for diagnosing TPE.

## Introduction

Tuberculosis (TB) is a global public threat caused by *Mycobacterium tuberculosis* (*Mtb*). In 2020, approximately 1.5 million people worldwide died from TB [1]. TB is categorized into pulmonary TB and extrapulmonary TB. Tuberculous pleurisy is one of the most common extrapulmonary TB, accounting for approximately 3% to 10% of all TB cases [2–4]. Pleural effusion (PE) is a common sign of tuberculous pleurisy, termed tuberculous PE (TPE). Although PE can be seen in nearly all patients with tuberculous pleurisy, it is not a specific sign. Malignant disease, heart failure and pneumonia can also cause PE [5, 6]. Identifying TPE in undiagnosed PE patients is crucial because the delayed diagnosis can result in pulmonary TB, bronchopleural fistulas, TB empyema, and even long-term functional impairment [4].

Pleural fluid (PF) Ziehl–Neelsen staining and culture are the gold standard for diagnosing TPE because of their high specificity; however, their sensitivities were unsatisfactory [6, 7]. Pleural biopsy, guided by imaging or thoracoscopy, is another gold standard for diagnosing TPE. However, it is invasive and can cause operating-related complications such as subcutaneous emphysema, fever and bleeding [8–11]. Nucleic acid amplification tests (NAATs), especially Xpert MTB/RIF, have been widely used for diagnosing TPE because of their high specificities; however, their sensitivities were around 30% and 50% [12–14]. Therefore, developing novel diagnostic tools for TPE is of great value.

PF biomarkers are complementary diagnostic tools for TPE because of their low cost, short turn-around time (TAT), and objectiveness [12]. Adenosine deaminase (ADA) is the most studied among all available PF biomarkers. According to the published meta-analyses, PF ADA has a sensitivity of 90% and specificity of 92% [15, 16]. Although PF ADA is not the gold standard test for the diagnosis of TPE, it is recommended as a ‘rule out’ test in countries with a low prevalence of TB, according to the British Thoracic Society Pleural Disease Guideline [17]. Given the imperfect diagnostic accuracy of ADA, it remains necessary to develop novel biomarkers to improve its diagnostic accuracy or replace it [12]. Recent studies showed that the PF lactate dehydrogenase (LDH) to ADA (LDH/ADA) ratio is another complementary tool for TPE diagnosis [18–20]. However, these studies did not analyze whether the diagnostic value of the LDH/ADA ratio was superior to that of ADA. In other words, it remains unknown whether the introduction of LDH can add diagnostic value to ADA. Therefore, we performed this study to investigate the diagnostic accuracy of ADA and LDH/ADA ratio for TPE. Furthermore, we investigated whether LDH/ADA ratio provides added diagnostic value to ADA. We reported the study following the Standards for Reporting of Diagnostic Accuracy Studies (STARD) guideline [21, 22].

## Material and methods

### Participants

The participants in this study were from two cohorts, the BUFF (Biomarkers for patients with Undiagnosed pleural eFFusion) cohort and the SIMPLE (a Study Investigating Markers in PLeural Effusion) cohort. The BUFF is an observational study with retrospective data collection. The patients admitted to the Affiliated Hospital of Inner Mongolia Medical University (AHIMMU) between July 2017 and July 2018 were included in this study. The inclusion criteria were patients with undiagnosed pleural effusion who received PF cell count during admission. The exclusion criteria were: (i) patients aged < 18 years old; (ii) patients transferred to the AHIMMU with a definitive diagnosis and the treatment had been initiated before PF collection; (iii) patients without a definitive diagnosis after discharging from the AHIMMU; (iv) patients with much missing value in PF or serum biochemistry.

The SIMPLE is a prospective, pre-registered and double-blind diagnostic study that aims to investigate the diagnostic accuracy of circulating and PF biomarkers in undiagnosed PE patients [23]. The study protocol of the SIMPLE has been introduced previously [23]. In short, the inclusion criteria were patients with undiagnosed PE who visited the AHIMMU between September 2018 and July 2021 (Hohhot cohort). The exclusion criteria were as follows: (1) age < 18 years; (2) with a history of a known disease that could cause PE during the last three months; (3) pregnancy; (4) with comorbidities that can prevent thoracocentesis (e.g., coagulation disorder, impaired liver and renal function); (5) PE developed during hospitalization. Their PF and serum specimens were collected and stored between -70 and -80 ℃. In addition, a hospital in Changshu (Jiangsu Province of China) participated in the SIMPLE study from June 2020 to July 2021 (Changshu Cohort). The inclusion and exclusion criteria, as well as reference standards, were identical in the Hohhot and Changshu cohorts.

The ethics committee of the AHIMMU approved the BUFF study (No: KY2021014) and waived the need of informed consent because of its retrospective nature. The ethics committees of the AHIMMU and the Affiliated Changshu Hospital of Xuzhou Medical University approved the SIMPLE study (No: 2018011 and 2020-KY-009). Informed consent was obtained from all participants in the SIMPLE study. Both the SIMPLE and BUFF studies were performed in accordance with the Declaration of Helsinki.

### Data collection and biomarker assay

The demographic characteristics, final diagnosis, and laboratory results were extracted from the patient's medical records in the SIMPLE and BUFF cohort. Only the first one was used for analysis when multiple laboratory tests were ordered. The diagnosis of TPE was made with microbiology findings (e.g., Ziehl–Neelsen staining, *Mtb* culture, NAAT), pleural biopsy or treatment response. The diagnosis of parapneumonic pleural effusion (PPE) was based on signs, symptoms, imaging, and treatment response to antibiotics. Malignant pleural effusion (MPE) was diagnosed with pleural cytology, pleural biopsy and identification of primary cancer. The diagnosis of heart failire (HF) was made based on signs, laboratory findings (e.g., N-terminal pro-B-type natriuretic peptide), symptoms and treatment response. In the BUFF study and the Hohhot cohort in the SIMPLE study, the LDH and ADA activities in PF were determined by the Beckman AU5831 analyzer. In the Changshu Cohort of the SIMPLE study, PF LDH and ADA activities were determined by the Siemens ADVIA 2400 analyzer.

### Statistical analysis

Kolmogorov–Smirnov test was used to determine the normal distribution of continuous data. Mann–Whitney U test or student’s t-test were used to compare continuous data according to their distribution. The Chi-square test was used to compare categorical data. We used the receiver operating characteristic (ROC) curve to estimate the diagnostic accuracy of ADA and LDH/ADA ratio for TPE. Area under the ROC curve (AUC), a global indicator of diagnostic accuracy independent of threshold, was compared with Delong's method [24]. We used the net reclassification improvement (NRI) and integrated discrimination improvement (IDI) to estimate the added value of the LDH/ADA ratio to ADA [25]. The decision curve analysis (DCA) was used to estimate the net benefit of ADA and LDH/ADA ratio [26]. All analyses were performed with R (version 4.0.5), and p < 0.05 was regarded as statistically significant. The packages used in R include CBCgrps [27], PredictABEL [28], rmda, pROC [29] and ggplot2.

## Results

### Characteristics of the participants

**Fig. 1:**
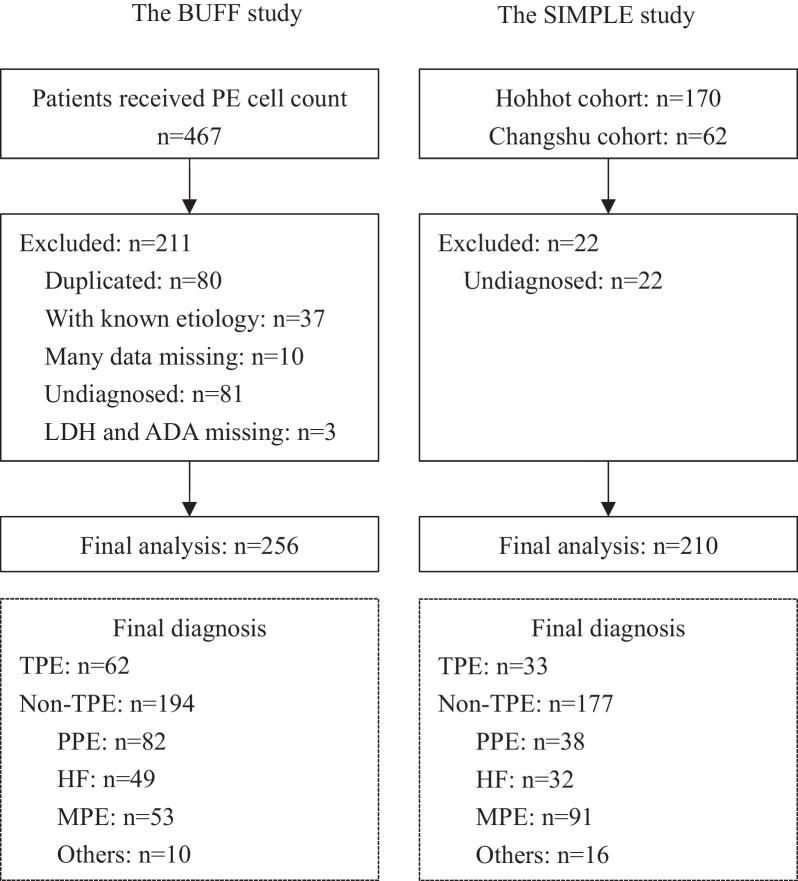
A flowchart of the patient selection process. HF, heart failure; MPE, malignant pleural effusion; PPE, parapneumonic effusions; TPE, tuberculous pleural effusion; LDH, fluid lactate dehydrogenase; ADA, adenosine deaminase

Figure 1 is a flowchart of the participants selection procedure. We included 256 patients in the BUFF and 210 patients in the SIMPLE study. The characteristics of the participants are listed in Table 1. In both the SIMPLE and the BUFF cohort, TPE patients had higher PF ADA activity, total protein concentration and lower LDH/ADA ratio than non-TPE patients. We failed to observe any differences in LDH activity between TPE and non-TPE patients.

**Table 1:** Demographic characteristics and pleural biochemistry of the participants

Characteristics	The BUFF cohort			The SIMPLE cohort		
	Non-TPE (n = 194)	TPE (n = 62)	p	Non-TPE (n = 177)	TPE (n = 33)	p
Sex, n (%)			0.080			0.539
Female	59 (30)	27 (44)		62 (35)	14 (42)	
Male	135 (70)	35 (56)		115 (65)	19 (58)	
Age, years	68 (58, 77)	68 (46, 76)	0.191	72 (65, 79)	72 (64, 79)	0.579
WBC, 10^6^/ml	878 (369, 2044)	1886 (933, 3108)	< 0.001	846 (416, 1829)	1574 (907, 2847)	< 0.001
Glucose, mmol/L	5.7 (4.3, 6.9)	4.6 (3.7, 6.2)	0.017	6.0 (4.9, 6.9)	5.3 (4.7, 6.4)	0.182
LDH, U/L	263 (119, 737)	367 (184, 685)	0.188	232 (143, 485)	283 (181, 390)	0.469
ADA, U/L	11 (6, 24)	35 (19, 43)	< 0.001	9 (5, 16)	42 (16, 53)	< 0.001
Protein, g/L	23 (16, 38)	34 (24, 43)	< 0.001	36 (23, 43)	44 (37, 47)	< 0.001

*WBC* white blood cell; *LDH* fluid lactate dehydrogenase; *ADA* adenosine deaminase

Continuous data were presented as the median and interquartile range, and compared with Mann–Whitney U test

### Diagnostic accuracy of PF ADA and LDH/ADA ratio

**Fig. 2:**
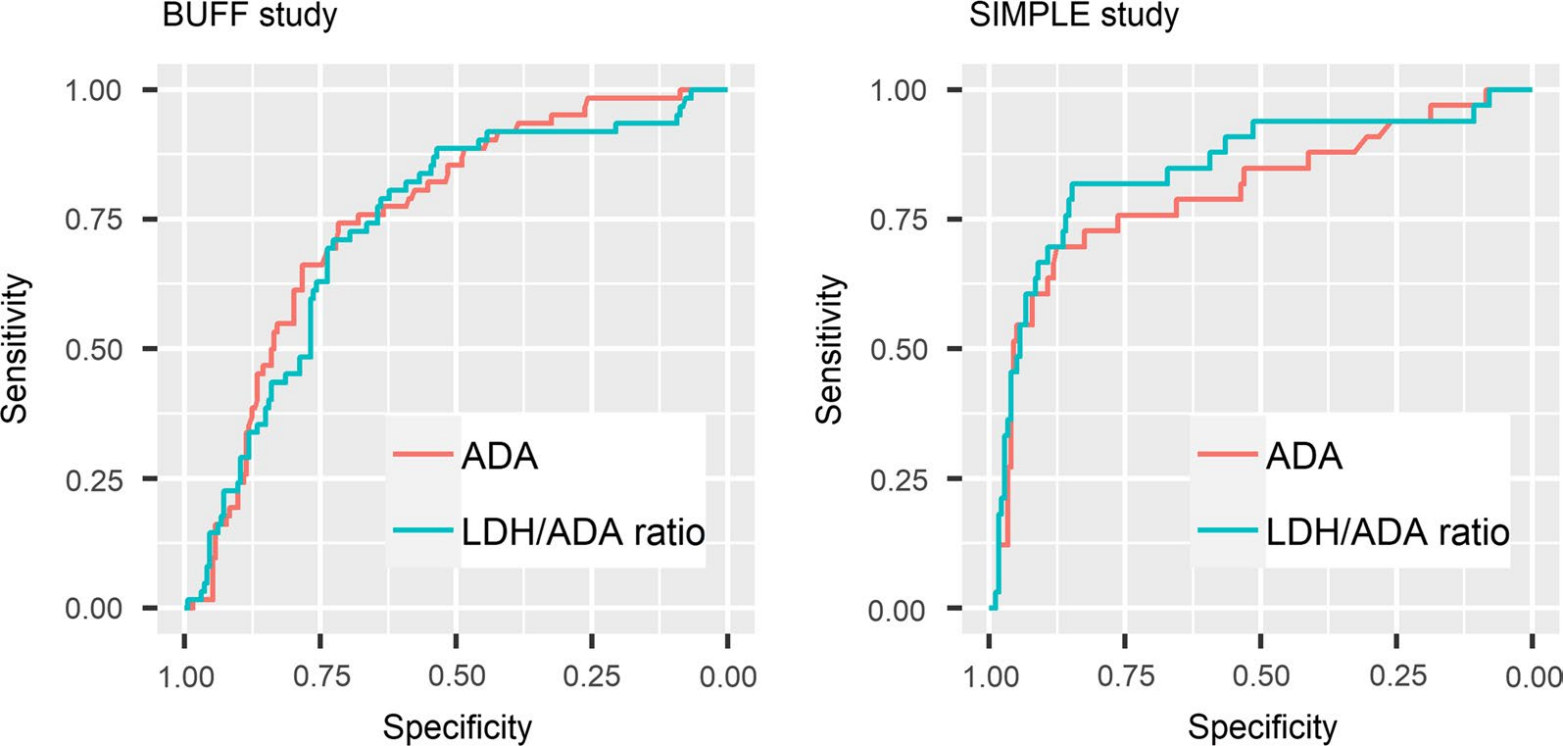
ROC curve of ADA and LDH/ADA ratio

**Fig. 3:**
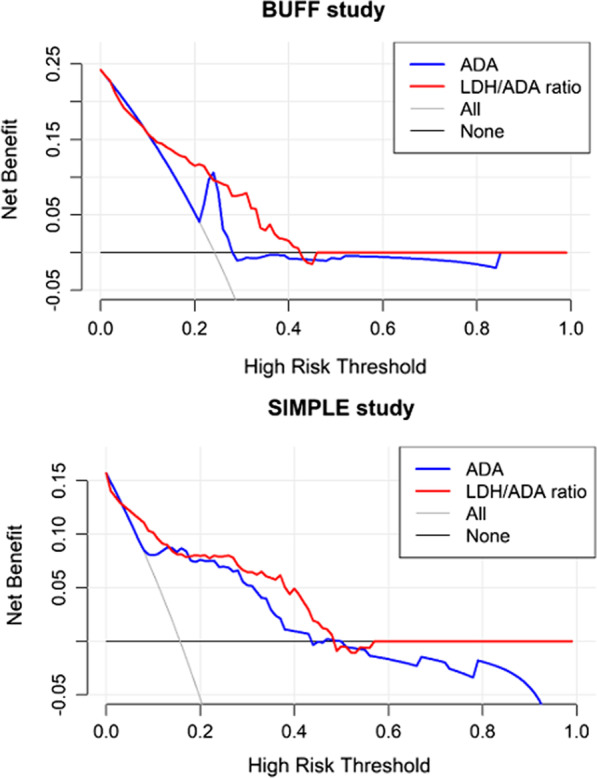
Decision curves of PF ADA and LDH/ADA ratio in the BUFF and SIMPLE cohorts

Figure 2 presents the ROC curves of PF ADA and LDH/ADA ratio. The AUCs and 95% confidence intervals (95% CIs) of ADA and LDH/ADA ratio in the BUFF cohort were 0.76 (95% CI: 0.69–0.82) and 0.74 (95% CI: 0.67–0.81), respectively (p = 0.67 for AUC comparison). In the SIMPLE cohort, the AUCs of ADA and LDH/ADA ratio were 0.80 (95% CI: 0.71–0.90) and 0.85 (95% CI: 0.76–0.93), respectively (p = 0.28 for AUC comparison). Therefore, the overall diagnostic accuracy of LDH/ADA does not outperform ADA. Figure 3 shows the decision curves of ADA and LDH/ADA. The LDH/ADA ratio did not show a significantly high net benefit over ADA in either the SIMPLE cohort or the BUFF cohort.

Table 2 lists the diagnostic accuracy of PF ADA and LDH/ADA ratio for TPE. Because the data-driven selection of the optimal threshold may overestimate the sensitivity and specificity of the testing investigated [30], we prespecified the thresholds of ADA and LDH/ADA ratio at 35 U/L and 20, respectively. In the BUFF cohort, the sensitivity and specificity of ADA were 0.47 (95% CI: 0.34–0.60) and 0.86 (95% CI: 0.80–0.90), respectively. The sensitivity and specificity of LDH/ADA ratio were 0.74 (95% CI: 0.63–0.85) and 0.66 (95% CI: 0.60–0.73), respectively. In the SIMPLE cohort, the sensitivity and specificity of ADA were 0.61 (95% CI: 0.45–0.76) and 0.92 (95% CI: 0.88–0.96), respectively. The sensitivity and specificity of ADA were 0.85 (95% CI: 0.70–0.97) and 0.67 (0.60–0.74), respectively.

**Table 2:** Diagnostic accuracy of PF ADA and LDH/ADA ratio

Testing	AUC (95% CI)	Threshold	Sensitivity (95% CI)	Specificity (95% CI)
*The BUFF study*				
LDH/ADA	0.74 (0.67–0.81)	20	0.74 (0.63–0.84)	0.66 (0.60–0.73)
ADA	0.76 (0.69–0.82)	35 U/L	0.47 (0.34–0.60)	0.86 (0.80–0.90)
*The SIMPLE study*				
LDH/ADA	0.85 (0.76–0.93)	20	0.85 (0.70–0.97)	0.67 (0.60–0.74)
ADA	0.80 (0.71–0.90)	35 U/L	0.61 (0.45–0.76)	0.92 (0.88–0.96)

Abbreviations have been explained in the footnote of Table 1

Table 3 lists the results of NRI and IDI analyses. We failed to observe any significant NRI and IDI (p > 0.05 for all) in both the SIMPLE and BUFF cohorts. In addition, we combined the BUFF and SIMPLE into a single cohort and calculated the NRI and IDI of the LDH/ADA ratio. Nevertheless, no statistically significant NRI and IDI were observed. These results indicate that PF LDH/ADA ratio does not provide added diagnostic value to ADA.

**Table 3:** Net reclassification improvement (NRI) and integrated discrimination improvement (IDI) analysis

Cohort	Continuous NRI		IDI	
	Estimates (95% CI)	p	Estimates (95% CI)	p
The BUFF study	− 0.23 (− 0.51–0.05)	0.104	− 0.016 (− 0.040–0.008)	0.185
The SIMPLE study	0.06 (− 0.30–0.43)	0.726	0.013 (− 0.036–0.062)	0.606
All	− 0.09 (− 0.32–0.13)	0.413	− 0.012 (− 0.035–0.011)	0.294

ADA was set as a reference, and the added diagnostic value of LDH/ADA was analyzed with IDI and continuous NRI

## Discussion

Although some previous studies have investigated the diagnostic accuracy of PF LDH/ADA ratio for TPE [18–20], they did not address the added value of LDH/ADA ratio to ADA. To the best of our knowledge, this is the first study investigating the added diagnostic value of the LDH/ADA ratio to ADA. With two cohorts, this study reveals that LDH/ADA ratio does not add incremental value to ADA for TPE diagnosis. Therefore, the current evidence does not support LDH/ADA ratio for diagnosing TPE.

To our knowledge, three studies have been performed to investigate the diagnostic value of the LDH/ADA ratio for TPE [18–20]. These studies did not compare the AUC of ADA and LDH/ADA ratio with statistical methods. Actually, the estimated AUC of ADA is slightly lower than that of the LDH/ADA ratio. In a study with a relatively larger sample size (n = 1637), the AUCs of ADA and LDH/ADA ratio are comparable (0.93 *vs.* 0.94), suggesting that LDH/ADA does not provide added diagnostic value beyond ADA. Unlike previous studies [18–20], we did not find a high diagnostic value for the LDH/ADA ratio. In both the BUFF and the SIMPLE cohorts, the AUC of LDH/ADA ratio was not statistically higher than that of ADA, suggesting the overall diagnostic accuracy of LDH/ADA ratio does not outperform ADA. The DCA also supported this conclusion. The decision curves of ADA and LDH/ADA ratio were very close in either the SIMPLE cohort or the BUFF cohort, indicating that LDH/ADA does not provide more benefits than ADA. Because the AUC of ROC has some limitations in estimating the overall diagnostic accuracy of a given test [31], we used the NRI and IDI to assess whether LDH/ADA ratio provides added diagnostic value beyond ADA. These two statistical methods are widely used in estimating added diagnostic value of a given diagnostic model [25]. IDI and continuous NRI of LDH/ADA ratio in the BUFF cohort were less than 0. In the SIMPLE cohort, although the IDI and the continuous NRI of LDH/ADA ratio were more than 0, the corresponding p values were less than 0.05. We thus concluded that LDH/ADA ratio does not improve the diagnostic accuracy of ADA. In other words, PF ADA is adequate for diagnosing TPE, and LDH is redundant. Indeed, the rationale for LDH/ADA as a diagnostic marker for TPE is that ADA increases in TPE patients while LDH decreases. Decreased LDH in TPE patients was observed in some studies [32–34]. However, this is not always true because some studies also reveal that PF LDH increases in TPE patients [35, 36]. Notably, in a study with large sample size and representative disease profile, only complicated parapneumonic effusion (CPPE) showed higher PF LDH activity than TPE patients [19, 37]. Patients with other causes, such as simple PPE and malignant pleural effusion, had comparable or lower LDH activity than that in TPE patients. Therefore, the diagnostic accuracy of PF LDH/ADA ratio may be greatly affected by the composition of controls, especially the portion and severity of PPE patients. This hypothesis is supported by the study performed by Vieira et al. [18]. In that study, only PPE patients were used as a control, and the AUC of LDH/ADA was higher than that of ADA (0.67 vs. 0.82).

Previous studies usually adopt 35 U/L as a threshold for TPE diagnosis [12, 15], which was also endorsed by the guideline [17]. Therefore, we used this threshold to determine the sensitivity and specificity of ADA in this study. The sensitivity and specificity of PF ADA in the BUFF cohort were 0.47 and 0.86, respectively. While in the SIMPLE cohort, the sensitivity and specificity were 0.61 and 0.92, respectively, which are lower than those reported by the previous studies and meta-analyses [15, 16]. This inconsistency can be partially explained by the age of participants in our study. The median ages of participants in the BUFF and the SIMPLE cohorts were 68 years and 72 years, respectively, which is higher than that in previous studies [15, 16]. Some previous studies also revealed that age could affect the diagnostic accuracy of PF ADA for TPE, and the diagnostic accuracy of ADA decreased in old patients [38, 39].

Although this is the first study investigating the added diagnostic value of PF LDH/ADA, it has some limitations. First, the retrospective nature of the BUFF cohort and the small sample size may bias the results. Second, the diagnosis of TPE and non-TPE was based on the different reference standards, termed differential verification bias [40]. This design weakness may overestimate the diagnostic accuracy of LDH/ADA ratio and ADA. However, differential verification bias is common in diagnostic test accuracy studies investigating PF biomarkers. Third, this study did not investigate factors affecting the diagnostic accuracy of ADA and LDH/ADA ratio because of the small sample size. Fourth, two ADA assays were used in this work. It is unclear whether the ADA assay can affect the ADA value and its diagnostic accuracy.

## Conclusions

In conclusion, we failed to find the added diagnostic value of LDH/ADA to ADA. Therefore, the current evidence does not support LDH/ADA for TPE diagnosis. Given the small sample size and potential bias, further rigorous studies with large sample sizes are needed to validate our findings.

## Data Availability

All data generated or analyzed during this study are included in this published article.

## Abbreviations

TB: Tuberculosis
*Mtb*: *Mycobacterium tuberculosis*
PE: Pleural effusion
TPE: Tuberculous pleural effusion
PF: Pleural fluid
NAATs: Nucleic acid amplification tests
ADA: Adenosine deaminase
LDH: Lactate dehydrogenase
LDH/ADA ratio: Lactate dehydrogenase to adenosine deaminase ratio
STARD: Standards for Reporting of Diagnostic Accuracy Studies
ROC: Receiver operating characteristic
AUC: Area under the ROC curve
NRI: Net reclassification improvement
IDI: Integrated discrimination improvement
DCA: Decision curve analysis
HF: Heart failure
MPE: Malignant pleural effusion
PPE: Parapneumonic effusions
WBC: White blood cell
CPPE: Complicated parapneumonic effusion
